# Incidence and Predictors of Left Atrial Appendage Thrombus before Catheter Ablation of Thrombogenic Arrhythmias

**DOI:** 10.3390/jpm12030460

**Published:** 2022-03-14

**Authors:** Felix K. Wegner, Robert M. Radke, Christian Ellermann, Julian Wolfes, Kevin Willy, Philipp S. Lange, Gerrit Frommeyer, Helmut Baumgartner, Lars Eckardt, Gerhard-Paul Diller, Stefan Orwat

**Affiliations:** 1 Department of Cardiology II—Electrophysiology, University Hospital Muenster, Albert Schweitzer Campus 1, 48149 Muenster, Germany; christian.ellermann@ukmuenster.de (C.E.); julian.wolfes@ukmuenster.de (J.W.); kevin.willy@ukmuenster.de (K.W.); philippsebastian.lange@ukmuenster.de (P.S.L.); gerrit.frommeyer@ukmuenster.de (G.F.); lars.eckardt@ukmuenster.de (L.E.); 2 Department of Cardiology III—Adult Congenital and Valvular Heart Disease, University Hospital Muenster, Albert Schweitzer Campus 1, 48149 Muenster, Germany; robert.radke@ukmuenster.de (R.M.R.); helmut.baumgartner@ukmuenster.de (H.B.); gerhard.diller@ukmuenster.de (G.-P.D.); stefan.orwat@ukmuenster.de (S.O.)

**Keywords:** left atrial appendage, thrombus, catheter ablation, atrial fibrillation, atrial flutter

## Abstract

Introduction: Transesophageal echocardiography (TEE) is routinely performed before catheter ablation of atrial tachyarrhythmias to rule out the presence of left atrial thrombi. However, data to support this practice are inconsistent. Methods: We analyzed consecutive pre-procedural TEE in a high-volume electrophysiology center for the presence of left atrial thrombi and a relevant flow reduction in the left atrial appendage (LAA) defined as LAA sludge or LAA emptying velocity (LAAEV) < 20 cm/s. The possible predictors of reduced flow were recorded and compared in a multivariate logistic regression analysis. Results: 1676 TEE were included (1122 before pulmonary vein isolation, 436 before atrial flutter ablation, 166 before other ablations). 543 patients (32%) were female and 991 (59%) were on DOAC. Nine patients (0.5%) had an LAA thrombus on pre-procedural TEE. Ninety-five further patients (5.7%) had a relevant reduction in LAA flow. The underlying rhythm showed a significant association with the presence of LAA thrombus or reduced LAA flow (*p* = 0.003). Patients in sinus rhythm and cavotricuspid isthmus-dependent atrial flutter exhibited the lowest risk. Additionally, reduced kidney function was associated with a reduction in LAA flow velocities (*p* = 0.04). Of note, two LAA thrombi occurred in patients in sinus rhythm and six out of nine patients with an LAA thrombus were on vitamin-K antagonists. Conclusions: LAA thrombus is a rare occurrence before an elective catheter ablation. The underlying rhythm and kidney function may serve as markers of a higher likelihood of significantly reduced LAAEV and LAA thrombus.

## 1. Introduction

Catheter ablation is an effective rhythm control strategy in atrial fibrillation with likely prognostic benefits [1,2,3]. The exclusion of a left atrial appendage (LAA) thrombus by transesophageal echocardiography (TEE) or cardiac computed tomography (CT) is part of routine pre-interventional workup to reduce the risk of embolic events such as stroke [4]. This also applies for other invasive electrophysiology (EP) studies in thrombogenic arrhythmias, such as cavotricuspid isthmus (CTI) or left atrial ablations in atrial flutter. However, there is considerable uncertainty regarding the exclusion of LAA thrombi with heterogeneous studies reporting pre-interventional incidences between 0.2–4.4% [5,6,7,8,9]. With mounting evidence of the prognostic benefits of early rhythm control in atrial fibrillation, the number of ablation procedures is expected to increase in the near future. Identifying patient populations which may be safely ablated without prior exclusion of a LAA thrombus may, therefore, reduce the possible complications associated with TEE or radiation exposure with cardiac CT, and optimize healthcare expenditure, especially in resource-limited settings. Therefore, the present study was designed to analyze the incidence of LAA thrombi before catheter ablation in a large cohort and to search for the predictive factors of low LAA blood flow velocities.

## 2. Materials and Methods

The present study was designed as a retrospective cohort study and conducted in accordance with the Declaration of Helsinki. All 1676 pre-interventional TEE before a catheter ablation in a large electrophysiology center from 2017–2020 were analyzed. Clinical characteristics were documented and analyzed in a multivariate logistic regression model to elicit possible predictive factors of low-flow situations in the LAA. Three patient groups were formed:

- Patients with a solid LAA thrombus on pre-interventional TEE.
- Patients with a low-flow situation of the LAA, defined as:
  - LAA sludge without evidence of a solid thrombus and/or
  - LAA emptying velocity (LAAEV) ≤ 20 cm/s on PW-Doppler.
- Patients with normal LAA flow characteristics and without a LAA thrombus.

### 2.1. Transesophageal Echocardiography

Transesophageal echocardiography was conducted according to guideline-recommended practice [10]. All patients gave informed consent for TEE and for the subsequent catheter ablation. After contraindications of TEE were excluded, patients were sedated with intravenous propofol. When adequate sedation was reached, the ultrasound probe was introduced and moved to a mid-esophageal position. Images of the LAA were obtained in at least two planes (60° and 120°) with and without color Doppler and blood flow velocity of the LAA was measured using pulsed-wave Doppler (PW). Additional planes, four-dimensional imaging and the measurement of tissue Doppler velocities were at the discretion of the performing physician. The ultrasound probe was withdrawn once adequate images were obtained and the patient was moved to the EP laboratory for ablation if no contraindications had been found.

### 2.2. Catheter Ablation

All patients without evidence of a solid LAA thrombus received the scheduled catheter ablation. Venous access was gained through the femoral veins and diagnostic catheters were placed. The underlying rhythm was determined by intracardiac electrogram and diagnostic maneuvers as necessary. For the purpose of this study, patients with atrial flutter were divided into two categories. If the cavotricuspid isthmus was part of the reentry circuit, the rhythm was defined as typical CTI-dependent atrial flutter. All other types of atrial flutter such as left atrial and scar-related flutter were classified as atypical atrial flutter. Subsequently, ablation catheters were placed and the ablation procedure was conducted according to established recommendations and usual clinical practice [4]. For pulmonary vein isolation (PVI) and other left atrial ablations, left atrial access was gained by a fluoroscopic-guided transseptal puncture. Patients receiving their first PVI were treated with the current-generation cryoballoon (Medtronic, Minneapolis, MN, USA).

### 2.3. Periprocedural Anticoagulation

Patients on vitamin-K antagonist anticoagulants (VKA) were instructed to aim for an international normalized ratio (INR) of 2.0–2.5 on the day of the planned ablation. In patients with an INR below 2.0, regular VKA dosage was increased to reach the therapeutic range as soon as possible without risking a supratherapeutic INR. For patients on direct oral anticoagulants (DOAC), the ablation was performed under continuous intake of the oral anticoagulation. All patients received intravenous heparin during the ablation procedure with regular intraprocedural controls of the activated clotting time.

### 2.4. Statistical Analysis

Data storage was conducted using Microsoft Excel© 2010 (Microsoft Corporation, Redmond, WA, USA) and SPSS Version 27 (IBM Corporation, Somers, NY, USA) was used for statistical analysis. Mann–Whitney U, chi-square or Fisher exact tests were used for univariate analyses as appropriate. A multivariate logistic regression model was utilized for multivariate analysis. Statistical significance was defined as a two-sided alpha level of 0.05 or less.

## 3. Results

In total, 1676 pre-interventional TEE studies were included in the analysis. Table 1 depicts the baseline characteristics of the patient population. 991 patients (59%) were on direct oral anticoagulants (DOAC), whereas 297 (18%) were on oral vitamin K antagonists (VKA). Median INR of the patients on VKA was 2.1 with an interquartile range (IQR) of 1.9–2.4. The median LVEF on pre-procedural transthoracic echocardiography was 60% (IQR 53–65%), median glomerular filtration rate (GFR, Cockroft–Gault formula) was 92 mL/min (IQR 71–116 mL/min) and the mean EHRA score of included patients was 2.4 ± 0.7. No severe complications occurred during any of the 1676 TEE studies. 228 patients exhibited a typical isthmus-dependent atrial flutter as determined by the subsequent EP study, whereas 105 patients had atypical atrial flutter. The group of patients with no anticoagulation on admission had a median age of 53 years (IQR 48–59), a median CHA_2_DS_2_-VASc score of 0 (IQR 0–1), a median BMI of 26 kg/m^2^ (IQR 24–28 kg/m^2^) and a median GFR of 112 mL/min (IQR 95–134 mL/min).

### 3.1. Incidence of LAA Thrombus

**Figure 1:**
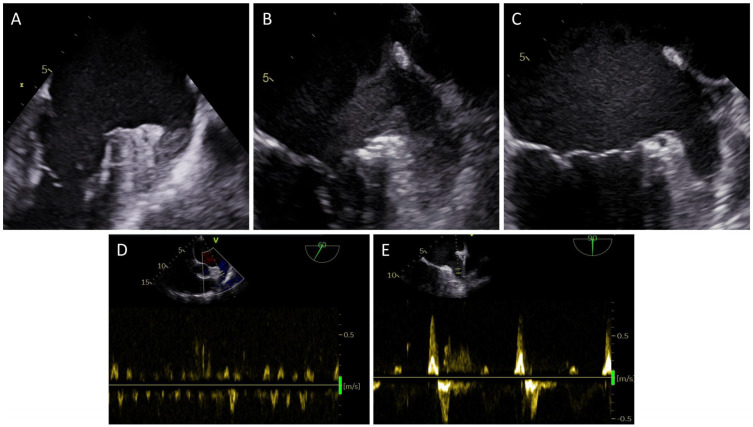
Depiction of a solid LAA thrombus (**A**), LAA sludge without evidence of a solid LAA thrombus on subsequent IV contrast application (**B**), an empty LAA (**C**), reduced LAA emptying velocity < 20 cm/s (**D**) and a normal LAA emptying velocity ≥ 50 cm/s (**E**).

Nine TEE studies (0.5%) depicted a solid thrombus of the LAA (see Figure 1). Table 2 contains the clinical characteristics of this patient group. Whereas five of nine patients were in atrial fibrillation during TEE, two patients with a LAA thrombus were in sinus rhythm. Both of these patients were on VKA at the time of TEE, with a subtherapeutic INR on admission of 1.5 and 1.8, respectively. In total, six of nine patients with an LAA thrombus were on VKA at the time of TEE. The median INR of these patients was 1.8 with an interquartile range of 1.7–1.9. Of note, one patient who exhibited an LAA thrombus had an INR of 3.5. The median LVEF of patients with an LAA thrombus was 32% and eight of nine patients had a reduced LAAEV of ≤20 cm/s in addition to the LAA thrombus. In all patients, the planned EP study was postponed. Following the diagnosis of an LAA thrombus, the further anticoagulation management was discussed with the patients on an individual basis after careful discussion of medication intake history and possible alternatives: two patients were switched from VKA to apixaban and one patient each was switched from VKA and acetylsalicylic acid (ASA) to dabigatran. One further patient was switched from edoxaban to VKA. One patient remained on apixaban, and three patients remained on VKA but were instructed on close medication adherence and INR monitoring.

### 3.2. Incidence of Reduced LAA Flow Velocity

Ninety-five patients (5.7%) exhibited a significantly reduced LAA emptying velocity of ≤20 cm/s and/or LAA sludge on pre-procedural TEE. Patient characteristics are shown in Table 2. Figure 1 shows representative images of LAA sludge (panel B) and reduced LAAEV <20 cm/s (panel D). In cases of large amounts of LAA sludge or when uncertainty persisted, IV ultrasound contrast was used to exclude a solid thrombus. After a solid thrombus was successfully excluded, all 95 patients underwent the scheduled EP study. There were no thromboembolic complications in these patients until hospital discharge.

### 3.3. Predictors of LAA Thrombus and Reduced LAA Flow

**Figure 2:**
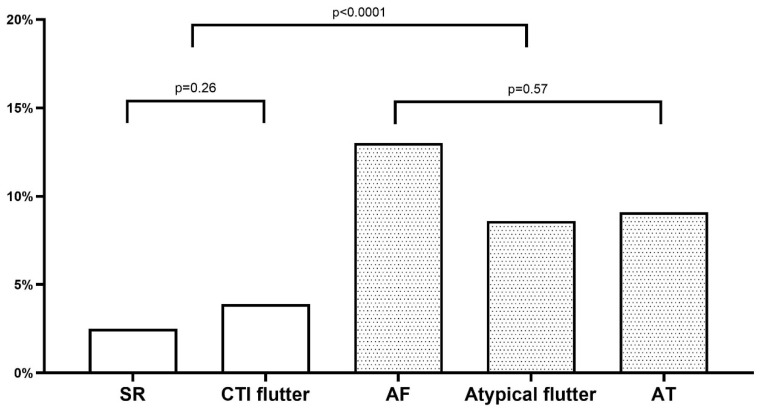
Proportion of patients with either a LAA thrombus or significantly reduced LAA emptying velocity stratified by underlying rhythm. AF = atrial fibrillation, AT = atrial tachycardia, CTI = cavotricuspid isthmus, SR = sinus rhythm.

We performed a multivariate logistic regression analysis to elicit possible predictors of LAA thrombi and reduced LAAEV. Patients with a LAA thrombus (*n* = 9) and with LAA sludge or severely reduced LAAEV (*n* = 95) were combined into one group of 104 patients for statistical analysis to offset unclear medication adherence and for statistical power. Univariate analyses showed a significant association between reduced LAA flow and possible predictors as shown in Table 2. Concerning the underlying rhythm at the time of TEE, two distinct risk categories were able to be identified (see Figure 2): patients in CTI-dependent atrial flutter had a low incidence of LAA thrombus or reduced LAAEV (3.9%) not statistically different from patients in sinus rhythm (2.5%; *p* = 0.26). However, patients in atrial fibrillation, atypical atrial flutter or atrial tachycardia constituted a group with a markedly increased incidence of LAA thrombus or reduced LAAEV (13%, 8.6% and 9.1%, respectively), which was highly significant on univariate analysis and remained significant in the multivariate logistic regression analysis (*p* = 0.003). Additionally, glomerular filtration rate (*p* = 0.04) was significantly associated with the presence of reduced LAA flow or LAA thrombus in the multivariate analysis (see Table 2). No further clinical characteristics showed a significant association with the occurrence of an LAA thrombus or reduced LAAEV on multivariate logistic regression analysis.

## 4. Discussion

The present study analyzed a large, contemporary cohort receiving pre-interventional TEE for the exclusion of LAA thrombus before atrial catheter ablation. We were able to document a very low incidence of LAA thrombus of 0.5% with an additional 5.7% of patients exhibiting a significantly reduced LAA emptying velocity.

In comparison with the available literature, we report a relatively low incidence of LAA thrombus similar to a recent report by Göldi et al. [5]. Since almost 60% of our patient population was on DOAC at the time of TEE, this might explain a reduction in the previously reported rate of LAA thrombus by studies mainly analyzing patients on VKA [8,9]. Additionally, the present study can support electrophysiologists to perform ablations despite LAA sludge or severely reduced LAA blood flow velocities in the absence of a solid LAA thrombus, as no thromboembolic complications occurred in this patient group.

Our data indicate a clinically relevant difference in the incidence of reduced LAA flow velocities according to the underlying rhythm at the time of TEE. Patients in sinus rhythm displayed the lowest incidence of reduced LAA blood flow. Interestingly, patients in typical, CTI-dependent atrial flutter at the time of TEE had only a slightly, non-significantly increased risk of reduced LAA flow velocity, whereas patients in atrial fibrillation, atypical flutter and atrial tachycardia had a markedly increased risk of either reduced LAAEV or LAA thrombus. This finding remained statistically significant on multivariate logistic regression analysis and is, therefore, unlikely to be confounded. A possible explanation may be that in sinus rhythm and CTI-dependent flutter, the left atrium is activated in a physiological, concentric manner conducive to an organized mechanical contraction of the LAA. Conversely, atrial fibrillation and atypical left atrial flutter electrically activate the LAA in a disorganized, high-frequency manner, whereas atrial tachycardia may indicate a high degree of atrial fibrosis. In this regard, the present study is the first to report a difference in the occurrence of reduced LAA flow velocities before catheter ablation according to atrial flutter type. Whereas our data would support forgoing TEE in patients in CTI-dependent flutter anticoagulated for ≥3 weeks analogous to the recommendations for patients in sinus rhythm [4], patients in atypical atrial flutter should continue to receive an exclusion of an LAA thrombus currently recommended for patients in atrial fibrillation.

Furthermore, we detected a significant association of kidney function and the presence of reduced LAA flow velocities and LAA thrombi. Whereas patients with chronic kidney disease have a higher risk of venous thromboembolism [11,12], kidney function has traditionally not been associated with thromboembolic events in atrial fibrillation and has not been included in risk scores such as the CHA_2_DS_2_-VASc score. Prospective studies are necessary to analyze whether these findings can be applied to all patients in thrombogenic arrhythmias.

While the anticoagulation regimen did not show an association with reduced LAA blood flow velocities, two thirds of patients in our cohort with a solid LAA thrombus were anticoagulated with VKA. This was despite the fact that the admission INR of these patients was only slightly out of the target range of 2.0–3.0. Therefore, it appears reasonable to evaluate patients with reduced LAAEV or LAA sludge who are on VKA for a transition to DOAC to avoid progression to a solid LAA thrombus. Additionally, the absence of oral anticoagulation on admission was not associated with an increase in the presence of reduced LAAEV or LAA thrombi. This was most likely due to this group of patients being comparatively healthier than the overall average of the included patients with a lower age, BMI and CHA_2_DS_2_-VASc score. Patients without indication for long-term anticoagulation, according to their CHA_2_DS_2_-VASc score, received intermittent anticoagulation after the ablation if either a cardioversion or a left atrial ablation was performed.

The CHA_2_DS_2_-VASc score did not show a statistically significant association with the presence of LAA thrombi or reduced LAA flow velocities on multivariate analysis in our patient population. Although this may indicate that other factors such as underlying rhythm had a higher discriminatory power in our comparatively healthy patient population, it should not be interpreted to suggest an irrelevance of the CHA_2_DS_2_-VASc score in the risk assessment for thromboembolic events. Additionally, patients with a LAA thrombus had a noticeably lower median LVEF compared to patients without LAA thrombi, indicating an important role of left ventricular function in patients at a high risk of the formation of LAA thrombi. Further precipitating factors for the development of AF, such as concomitant hyperthyroidism [13] or the presence of cardiac channelopathies [14,15,16], did not show an association with the presence of reduced LAAEV or LAA thrombus in our patient population. Data on additional possible predictors such as endurance sports [17], alcohol consumption or smoking [18] was not routinely collected and, therefore, not available for analysis.

In our center, TEE is routinely performed before catheter ablation as it has a very low incidence of complications and does not confer the risks of ionizing radiation associated with CT [19,20,21,22]. Cardiac CT was reserved for specific circumstances such as equivocal TEE findings, an inability to perform TEE or additional pre-procedural characterization of pulmonary veins in select cases.

## 5. Limitations

Although the present study reports on a contemporary patient population, its retrospective and single-center design result in limitations concerning the generalizability of its findings. Medication adherence before catheter ablation was not systematically monitored and thus no association could be documented between the time outside of therapeutic INR (in case of VKA) or the number of missed doses (in case of DOAC) and the occurrence of LAA thrombi. However, LAAEV was not influenced by the choice of anticoagulation and, therefore, the analysis of possible predictors of reduced blood flow velocities was unlikely to be affected.

## 6. Conclusions

The presence of a LAA thrombus is a very rare occurrence before elective catheter ablation in thrombogenic arrhythmias. Patients in sinus rhythm and cavotricuspid isthmus-dependent atrial flutter at the time of ablation as well as patients with preserved kidney function exhibited the lowest risk for the presence of LAA thrombi.

## Figures and Tables

**Table 1:** Baseline characteristics of all patients.

Gender Male/Female	1133/543 (68%/32%)
Age in years (IQR)	63 (56–73)
BMI in kg/m^2^ (IQR)	27 (25–30)
Underlying rhythm – Sinus rhythm – Atrial fibrillation – CTI flutter – Atypical flutter – Atrial tachycardia	815 (49%) 481 (29%) 230 (14%) 105 (6%) 45 (2%)
CHA_2_DS_2_-VASc score – 0 – 1 – 2 – 3 – 4 – ≥5	268 (16%) 384 (23%) 387 (23%) 302 (18%) 201 (12%) 134 (8%)
Cardiovascular comorbidities – Hypertension – Coronary heart disease – Diabetes – Previous stroke – Peripheral artery disease	877 (52%) 179 (11%) 151 (9%) 52 (3%) 45 (3%)
Anticoagulation regimen – VKA – Apixaban – Rivaroxaban – Edoxaban – Dabigatran – ASA – None	306 (18%) 519 (31%) 203 (12%) 224 (13%) 45 (3%) 33 (2%) 346 (21%)

For age and BMI, the median is given. ASA = acetylsalicylic acid, IQR = interquartile range, VKA = vitamin K antagonist.

**Table 2:** Comparison between patients with normal LAA flow, patients with significantly reduced flow and patients with a LAA thrombus.

	Normal LAA Flow (*n* = 1572)	Reduced LAA Flow (*n* = 95)	LAA Thrombus (*n* = 9)	*p*-Value (Univariate)	*p*-Value (Multivariate)
Gender (male/female)	1074 (68%)/498 (32%)	53 (56%)/ 42 (44%)	6 (67%)/ 3 (33%)	0.02	0.30
Age in years (IQR)	63 (55–72)	71 (62–77)	77 (51–79)	<0.0001	0.94
BMI in kg/m^2^ (IQR)	27 (25–30)	27 (24–31)	28 (26–30)	0.58	
Underlying rhythm – Sinus rhythm – Atrial fibrillation – CTI flutter – Atypical flutter – AT	795 (51%) 420 (27%) 221 (14%) 96 (6%) 40 (3%)	18 (19%) 56 (59%) 8 (8%) 9 (9%) 4 (4%)	2 (22%) 5 (56%) 1 (11%) 0 1 (11%)	<0.0001	**0.003**
CHA_2_DS_2_-VASc score	2.0 ± 1.5	3.2 ± 1.5	3.8 ± 2.0	<0.0001	0.38
EHRA score	2.4 ± 0.7	2.3 ± 0.8	2.8 ± 0.5	0.40	
Coronary heart disease	160 (10%)	15 (16%)	4 (44%)	0.01	0.94
Diabetes	142 (9%)	7 (7%)	2 (22%)	0.90	
Hypertension	808 (51%)	63 (66%)	6 (67%)	<0.01	0.63
Previous stroke	45 (3%)	6 (6%)	1 (11%)	0.03	0.14
Peripheral artery disease	36 (2%)	7 (7%)	2 (22%)	<0.01	0.30
Anticoagulation – DOAC – VKA – ASA – None	931 (59%) 270 (17%) 30 (2%) 341 (22%)	58 (61%) 29 (31%) 2 (2%) 5 (5%)	2 (22%) 6 (67%) 1 (11%) 0	<0.001	0.10
Heart rate in 1/min (IQR)	71 (60–95)	84 (70–103)	75 (56–99)	<0.01	0.63
QRS width in ms (IQR)	100 (90–110)	100 (90–120)	110 (90–150)	<0.01	0.20
LVEF in % (IQR)	60 (55–60)	53 (45–60)	32 (22–49)	<0.0001	0.55
GFR in ml/min (IQR)	94 (73–117)	69 (56–91)	80 (34–150)	<0.0001	**0.04**
Atrial fibrillation state – Paroxysmal – Persistent – Permanent	708 (45%) 833 (53%) 31 (2%)	38 (40%) 54 (57%) 3 (3%)	2 (22%) 7 (78%) 0	<0.001	0.20
Channelopathies	7 (<1%)	1 (1%)	0	0.99	
Hyperthyroidism	49 (3%)	3 (3%)	0	0.24	

Statistical significance was calculated between the group with normal flow and a combination of the other two groups (*n* = 104) in a multivariate logistic regression analysis. Channelopathies include short QT syndrome, long QT syndrome, Brugada syndrome and catecholaminergic polymorphic ventricular tachycardia. Statistically significant results are displayed in bold. ASA = acetylsalicylic acid, AT = atrial tachycardia, IQR = interquartile range, LMWH = low-molecular-weight heparin.

## Data Availability

The data used for the current study are available from the corresponding author on reasonable request.
